# SERS-Based Evaluation of the DNA Methylation Pattern Associated With Progression in Clonal Leukemogenesis of Down Syndrome

**DOI:** 10.3389/fbioe.2021.703268

**Published:** 2021-07-23

**Authors:** Vlad Moisoiu, Valentina Sas, Andrei Stefancu, Stefania D. Iancu, Ancuta Jurj, Sergiu Pasca, Sabina Iluta, Alina-Andreea Zimta, Adrian B. Tigu, Patric Teodorescu, Cristina Turcas, Cristina Blag, Delia Dima, Gheorghe Popa, Smaranda Arghirescu, Sorin Man, Anca Colita, Nicolae Leopold, Ciprian Tomuleasa

**Affiliations:** ^1^Faculty of Physics, Babeş-Bolyai University, Cluj-Napoca, Romania; ^2^Department of Hematology, Iuliu Hatieganu University of Medicine and Pharmacy, Cluj-Napoca, Romania; ^3^Department of Pediatrics, Iuliu Hatieganu University of Medicine and Pharmacy, Cluj-Napoca, Romania; ^4^Research Center for Functional Genomics and Translational Medicine, Iuliu Hatieganu University of Medicine and Pharmacy, Cluj-Napoca, Romania; ^5^Medfuture Research Center for Advanced Medicine, Iuliu Hatieganu University of Medicine and Pharmacy, Cluj-Napoca, Romania; ^6^Department of Pediatrics, Victor Babeş University of Medicine and Pharmacy, Timisoara, Romania; ^7^Department of Pediatrics, Louis Turcanu Emergency Hospital for Children, Timisoara, Romania; ^8^Department of Pediatrics, Carol Davila University of Medicine and Pharmacy, Bucharest, Romania; ^9^Department of Pediatrics, Fundeni Clinical Institute, Bucharest, Romania; ^10^Biomed Data Analytics SRL, Cluj-Napoca, Romania; ^11^Department of Hematology, Ion Chiricuta Clinical Cancer Center, Cluj-Napoca, Romania

**Keywords:** SERS, down syndrome, acute leukemia, transient leukemia associated with down syndrome, myeloproliferative neoplasm

## Abstract

Here we show that surface-enhanced Raman scattering (SERS) analysis captures the relative hypomethylation of DNA from patients with acute leukemia associated with Down syndrome (AL-DS) compared with patients diagnosed with transient leukemia associated with Down syndrome (TL-DS), an information inferred from the area under the SERS band at 1005 cm^–1^ attributed to 5-methycytosine. The receiver operating characteristic (ROC) analysis of the area under the SERS band at 1005 cm^–1^ yielded an area under the curve (AUC) of 0.77 in differentiating between the AL-DS and TL-DS groups. In addition, we showed that DNA from patients with non-DS myeloproliferative neoplasm (non-DS-MPN) is hypomethylated compared to non-DS-AL, the area under the SERS band at 1005 cm^–1^ yielding an AUC of 0.78 in separating between non-DS-MPN and non-DS-AL. Overall, in this study, the area of the 1005 cm^–1^ DNA SERS marker band shows a stepwise decrease in DNA global methylation as cells progress from a pre-leukemia to a full-blown acute leukemia, highlighting thus the potential of SERS as an emerging method of analyzing the methylation landscape of DNA in the context of leukemia genesis and progression.

## Introduction

DNA methylation (along with other epigenetic modifications) is a key factor contributing to the audacity with which cancer cell resist to any form of treatment (Galle et al., 2020). Indeed, mounting evidence suggests that epigenetic changes orchestrate the phenotypic plasticity which allows cancer cells to escape virtually any form of treatment, be it chemotherapy, radiotherapy, surgery, or immunotherapy (Flavahan et al., 2017; McMahon et al., 2017; Zhu et al., 2018). Thus, it is believed that a better understanding of the epigenetic mechanisms behind cancer onset and progression is paramount for developing novel cancer treatment strategies (Yang et al., 2010). However, efficient ways of studying this aberrant methylation landscape are still lacking.

Surface-enhanced Raman scattering (SERS) is a type of molecular spectroscopy technique which employs metal nanosubstrates for amplifying the Raman signal of molecules (Pilot et al., 2019). A key process in the amplification of the Raman signal is the adsorption (either physically or chemically) of the molecule onto the metal surface (Xie et al., 2020). Given that the unique methylation landscape of cancer DNA has been recently described to drive its preferential self-assembly onto metal surfaces (Sina et al., 2018), we thought that this phenomenon can be exploited for analyzing the cancer-associated DNA methylation landscape based on SERS. Starting from this hypothesis, we have recently demonstrated that SERS spectra of DNA from patients with acute leukemia (AL) exhibits a decreased intensity in the SERS band at 1005 cm^–1^ attributed to 5-methylcytosine compared to the DNA of control subjects, allowing a rapid and efficient differentiation between the two groups (Moisoiu et al., 2019). The results suggested that SERS analysis captures the cancer-associated global hypomethylation of DNA, which was one of the first cancer-specific epigenetic change described back in 1983 (Feinberg and Vogelstein, 1983a; Gama-Sosa et al., 1983). To the best of our knowledge, in the case of genomic DNA, methylation is the only epigenetic change that can be detected by SERS. Other epigenetic changes of DNA bases previously described in cancer (acetylation, hydroxymethylation, etc.) have been reported only for synthetic DNA bases (Barhoumi and Halas, 2011).

Building on these results, we focused in this study on patients with Down syndrome (DS) diagnosed with acute leukemia [termed acute leukemia associated with DS (AL-DS)] (Xavier et al., 2009). We chose this pathology since it was demonstrated that the DNA of patients with AL-DS exhibits a characteristic global hypomethylation, making it an ideal testbed for the SERS platform (Malinge et al., 2013). For comparison, we analyzed DS patients that developed a form of pre-leukemia termed transient leukemia associated with DS (TL-DS), an entity which was previously shown to exhibit hypermethylated DNA (Malinge et al., 2013). The fact that DNA from AL-DS cells is hypomethylated compared to the DNA from TL-DS cells represents an excellent opportunity for demonstrating the potential of SERS in probing the global methylation landscape.

In addition, we were also interested in SERS spectral differences between pre-leukemic and leukemic conditions outside the setting of DS. To this end, we included patients with myeloproliferative neoplasms (MPN) (Tefferi and Pardanani, 2015), which is a well characterized form of pre-leukemia, as well as patients with AL in our analysis. To distinguish these from patients with DS, we termed these groups as non-DS-MPN and non-DS-AL, respectively.

## Materials and Methods

### Patients

The patient cohort included 8 patients with AL-DS, 8 patients with TL-DS, 9 patients with non-DS-MPN and 5 patients with non-DS-AL. The study was approved by the Institutional Review Board of both the Iuliu Hatieganu University of Medicine and Pharmacy, as well as of the Emergency Clinical Children’s Hospital, both in Cluj-Napoca, Romania.

### DNA Extraction

All chemicals used in this study were of analytical grade. The DNA was extracted from archived bone marrow blood smear slides. Each slide was washed with 10 μL of phosphate buffer saline (PBS) followed by vigorous scrapping. All the material from the slide was transferred to a tube containing 190 μL of PBS. DNA was extracted using the QIAamp DNA Blood Mini kit for DNA extraction with minor modifications to the manufacturer’s protocol. Each sample was incubated with 20 μL of RNase R (Purelink, Thermo Fisher Scientific) at 56°C for 30 min instead of the 10 min recommendation of the manufacturer. The DNA was eluted in 40 μL of elution buffer for genomic DNA (AE buffer). The DNA samples were quantified using a NanoDrop UV-Vis absorption microvolume spectrometer (Thermo Fisher Scientific), yielding concentrations ranging between 1.4 and 16.8 ng/μL, with 260/280 ratio ranging from 1.99 to 6.61.

### SERS Analysis

Silver nanoparticles (AgNPs) synthesized by reduction of Ag^+^ with hydroxylamine hydrochloride were employed as SERS substrate, as previously described (Leopold and Lendl, 2003). Briefly, 17 mg of AgNO_3_ was dissolved in 90 mL ultrapure water (Millipore) under stirring. Separately, 17 mg of hydroxylamine hydrochloride was dissolved in 8.8 mL of ultrapure water, followed by the addition of 1.2 mL of NaOH 1% to the hydroxylamine solution. Finally, this mixture was added rapidly to the AgNO_3_ solution under stirring, the synthesis of AgNPs being indicated by an immediate change in color to brown-yellow. The silver colloidal solution was stored at room temperature. The stability of hya-AgNPs was determined by Zeta Potential measurements, the NPs Zeta Potential being −30.1 ± 8.53 mV (Supplementary Figure 1).

For sample preparation, 2 mL of the AgNPs were washed by centrifugation for 15 min at 7300 *g* and resuspended in 2 mL of ultrapure water. 8 μL of AgNPs were mixed with 2 μL of extracted DNA and with 0.5 μL of Ca(NO_3_)_2_ (final concentration 5 × 10^–4^ M). The mixture was vortexed for 30 s to ensure the adsorption of DNA to the AgNPs surface, and 5 μL from this mixture was placed on an aluminum foil covered microscope slide for SERS investigation. DNA adsorption was checked by fluorescence measurements (Supplementary Figure 2). Sybr Green fluorophore was used for DNA labeling. The fluorescence spectra were acquired by excitation with 442 nm laser line, which fits the absorption region of Sybr green.

The reproducibility of DNA SERS spectra was tested on DNA (150 ng/μl) extracted from a human cell line (HaCaT) using a similar protocol as above (Supplementary Figure 3). The SERS spectra were acquired during an 8 h period from 8 different mixtures of DNA and hya-AgNPs activated with Ca^2+^.

All SERS measurements were performed in liquid drop form by using the same batch of nanoparticles. SERS spectra were collected using an InVia Raman (Renishaw) spectrometer. For excitation, a doubled frequency Nd:YAG laser emitting at 532 nm was focused on the liquid drop through a 5X objective (Leica, NA 0.12), the laser power on the sample being approximately 25 mW. For each measurement an integration time of 40 s was set and three measurements were averaged.

### Statistical Analysis

Principal component analysis (PCA) is an unsupervised multivariate technique which reduces the dimensionality of the dataset. PCA yields a set of scores (represented as score plots) and corresponding principal component vectors (represented as loading plots). The loading plots highlight the SERS bands that have a significant variation across the dataset. PCA analysis was performed using The Unscrambler X (Camo Software). The first 7 PCs were used in all PCA models. The relation between the number of PCs and explained variance is depicted in Supplementary Figure 4.

For the univariate analysis, the area under the 1005 cm^–1^ SERS band was determined using custom built MATLAB R2019 scripts. The normality of the dataset was assessed using D’Agostino and Pearson omnibus normality test. The differences between the mean of datasets was assessed by Student’s *t* test (for parametric distributions) and Mann-Whitney *U* test (for non-parametric distributions), followed by the receiver operating characteristic (ROC) analysis. For all statistical analysis, the background was removed from the raw SERS spectra by applying a linear transformation, followed by the normalization to unitary area. All univariate statistical analysis was performed using Prism 6 (GraphPad).

## Results

**FIGURE 1:**
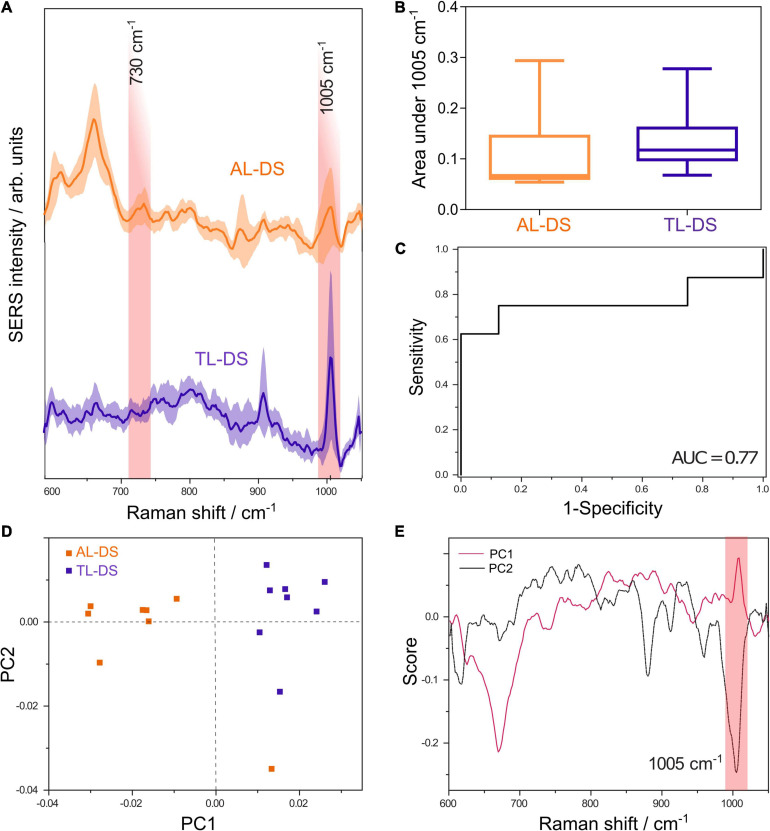
**(A)** Average SERS spectra of DNA from patients with acute leukemia associated with Down syndrome (AL-DS) and transient leukemia associated with Down syndrome (TL-DS). **(B)** The average normalized area under the SERS band at 1005 cm^– 1^ attributed to 5-methylcytosine was lower for the AL-DS group compared to the TL-DS group (one tailed Mann-Whitney *U* test, *p* = 0.04). **(C)** ROC curve for differentiating between AL-DS and TL-DS based on the normalized area under the 1005 cm^– 1^ SERS band, yielding an AUC of 0.77 (*p* = 0.07). **(D)** Score plot showing principal component (PC) 1 and PC2 resulted from the principal component analysis (PCA) of SERS spectra from AL-DS and TL-DS groups. **(E)** Loading plots corresponding to PC1 and PC2 from the PCA of SERS spectra from AL-DS and TL-DS groups.

The average SERS spectra of DNA from patients with AL-DS and TL-DS in the 600-1050 cm^–1^ range which comprises the most important SERS features of DNA, are shown in Figure 1A. The SERS spectra are dominated by the SERS band at 1005 cm^–1^ attributed to the rocking vibration of 5-methylcytosine (Moisoiu et al., 2019; Śanchez-Cortés and Garcia-Ramos, 1992), the SERS band at 730 cm^–1^ tentatively assigned to adenine (Garcia-Rico et al., 2018), the SERS band at 680 cm^–1^ tentatively assigned to guanine (Garcia-Rico et al., 2018), and the bands at 880 and 920 cm^–1^ attributed to the DNA backbone (Duguid et al., 1993). The SERS spectrum of the elution buffer was used as negative control, but no SERS bands were observed in 600–1050 cm^–1^ range (Supplementary Figure 5). As positive control, standard genomic DNA was used. The same spectral features as for the extracted DNA were observed in the positive control sample (Supplementary Figure 6).

As discussed in the section “Introduction,” the area of the 1005 cm^–1^ SERS band allows us to probe the relative hypomethylation of malignant DNA compared to non-malignant DNA (Moisoiu et al., 2019). Importantly, when comparing SERS spectra of cytosine, 5-methylcytosine, 1-methylcytosine, 1,5-dimethylcytosine, and 5-methylcytosine is the only one exhibiting a band around 1000 cm^–1^ (Śanchez-Cortés and Garcia-Ramos, 1992). The difference between the SERS spectrum of 5-methylcytosine alone (band around 1000 cm^–1)^ and the SERS spectrum of 5-cytosine methylated DNA (SERS band around 1005 cm^–1^) is expected, given that vibrational states are influenced by the global molecular structure.

To assess the possibility to discriminate between the two types of genomic DNA (AL-DS *vs.* TL-DS) based on univariate analysis of the 1005 cm^–1^ SERS band, we employed the area under the 1005 cm^–1^ band. The area under the SERS band at 1005 cm^–1^, informative of the methylation status of DNA, had a non-parametric distribution in both the AL-DS group (D’Agostino and Pearson omnibus normality test, *p* = 0.01) and the TL-DS group (D’Agostino and Pearson omnibus normality test, *p* = 0.01). Figure 1B shows that the average area under the SERS band at 1005 cm^–1^ was lower in AL-DS patients compared to TL-DS (one tailed Mann-Whitney *U* test, *p* = 0.04). This finding suggests that DNA from AL-DS patients is relatively hypomethylated compared to the DNA from patients with TL-DS. To quantify the difference, we calculated the receiver operating characteristic (ROC) for differentiating between the AL-DS and TL-DS groups, yielding an area under the curve (AUC) of 0.77 (*p* = 0.07) (Figure 1C).

Principal component analysis of the DNA SERS spectra highlights the unsupervised clustering of the AL-DS and TL-DS groups. Although PCA is not a classification algorithm, it shows whether the cohorts tend to cluster together based on the SERS spectra, indicating whether the groups can be classified accurately. Moreover, the relation between the score plot and loadings plot helped us pinpoint exactly what SERS spectral features explained the most variance of the whole dataset. Particularly, in our case, the PC1 axis showed a clear separation of the two groups, TL-DS and AL-DS (Figure 1D). The visual inspection of the PC1 loading plot showed an intense positive band at 1005 cm^–1^ (Figure 1E), which demonstrates that this band varied significantly across the dataset and contributed to the separation between the TL-DS and AL-DS groups. Importantly, the TL-DS group clustered toward the positive end of the PC1 score axis, while the AL-DS group clustered toward the negative end of the axis (Figure 1D), a distribution which should be correlated with the positivity of the band at 1005 cm^–1^ in the loading plot of PC1. Thus, PCA suggests that SERS spectra of DNA from AL-DS can be distinguished from TL-DS by a decrease in the intensity of the SERS band at 1005 cm^–1^, which is also what the univariate classification analysis proved (Figure 1C).

**FIGURE 2:**
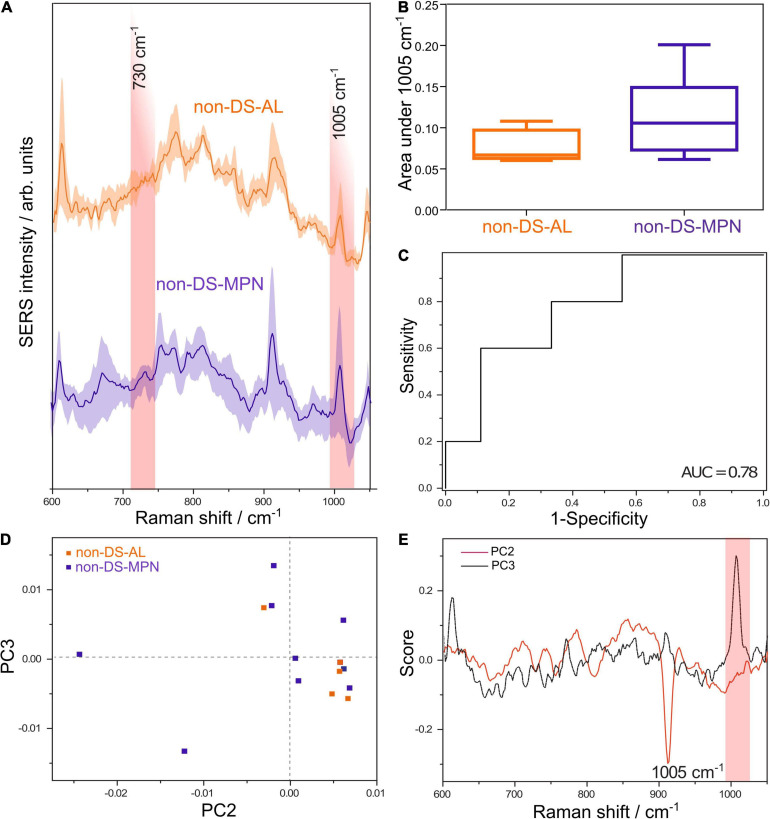
**(A)** Average SERS spectra of DNA from patients with non-DS-AL and non-DS-MPN. **(B)** The average normalized area under the SERS band at 1005 cm^– 1^ attributed to 5-methylcytosine is lower in the non-DS-AL group compared with the non-DS-MPN (one tailed Mann-Whitney *U* test, *p* = 0.05). **(C)** ROC curve for differentiating between non-DS-AL and non-DS-MPN based on the area of the 1005 cm^– 1^ SERS band, yielding an AUC of 0.78 (*p* = 0.09). **(D)** Score plot of the principal component analysis (PCA) of SERS spectra from non-DS-AL and non-DS-MPN groups. **(E)** Loading plots corresponding to PC2 and PC3 from the PCA of SERS spectra from non-DS-AL and non-DS-MPN groups.

To further explore whether the 1005 cm^–1^ SERS band can yield such significant results in other malignant DNA types, we compared DNA from patients with non-DS-AL and patients with non-DS-MPN. The average SERS spectra of DNA from patients with non-DS AL and non-DS-MPN are shown in Figure 2A. The area under the SERS band at 1005 cm^–1^ had a parametric distribution in the case of the non-DS-MPN group (D’Agostino and Pearson omnibus normality test, *p* = 1.10), while in the case of the non-DS-AL, the number of samples was too low for running the test. Figure 2B shows that similar to the case of AL-DS and TL-DS, the area under the SERS band at 1005 cm^–1^ is lower in the case of the non-DS-AL patients (a full-blown leukemia) compared to non-DS-MPN (a pre-leukemic condition) (one tailed Mann-Whitney *U* test, *p* = 0.05). The ROC analysis of the area under the SERS band at 1005 cm^–1^ yielded an AUC of 0.78 in discriminating between non-DS-AL *versus* non-DS-MPN (Figure 2C) (*p* = 0.09).

Principal component analysis of the SERS spectra of DNA from non-DS-AL and non-DS-MPN patients shows a tendency toward the unsupervised clustering of the non-DS-AL in the right lower quadrant (Figure 2D), corresponding to the positive end of the PC2 axis, and negative values on the PC3 axis. Correlated with the intense band at 1005 cm^–1^ in the loading plot of PC3 (Figure 2E), the clustering of the non-DS-AL samples at negative values on the PC3 axis, suggests that decreased intensities in the SERS band at 1005 cm^–1^ is a key feature contributing to the clustering of the non-DS-AL group. The relation between the number of PCs and explained variance is depicted in Supplementary Figure 7.

## Discussion

The methylation landscape of cancer DNA differs from its normal counterparts in at least two ways. Quantitatively, most cancer types exhibit global hypomethylation (Ehrlich, 2009; Feinberg and Vogelstein, 1983b; Gama-Sosa et al., 1983). Qualitatively, cancer DNA exhibits a variegated pattern of hypermethylated regions (the CpG islands found in the promoters of genes) alternating with regions devoid of 5-methylcytosine (Ehrlich, 2002). This pattern has been demonstrated to increase the affinity of cancer DNA for the surface of metal nanoparticles, a feature that can be exploited for SERS detection (Sina et al., 2018). We recently demonstrated that the hypomethylation characteristic to AL patients is apparent in the SERS spectra of peripheral blood genomic DNA as a decrease in the intensity of the SERS band at 1005 cm^–1^ attributed to 5-methylcytosine compared to control subjects (Moisoiu et al., 2019).

In this study, we extended these observations by comparing DNA from patients with full blown AL and pre-leukemic conditions in two settings: DS-associated malignancies and non-DS malignancies. The results suggest that DNA from AL-DS patients is hypomethylated compared to TL-DS (Figure 1B) (Mann-Whitney *U* test, *p* = 0.04), an information inferred from the area under the SERS band at 1005 cm^–1^ attributed to 5-methylcytosine. These results are in line with the study by Malinge et al., who reported that AL-DS cells exhibit a hypomethylated DNA (Malinge et al., 2013). In addition, we also presented data suggesting that DNA from non-DS-AL cells is hypomethylated compared with non-DS-MPN (Figure 2B). These results point toward a stepwise decrease in DNA methylation as cells progress from a normal state to pre-leukemia to a full-blown acute leukemia. In addition, this gradual decrease in DNA methylation occurs in both DS-associated malignancies and in patients without DS.

Similar data was also presented by Yun Yu et al., who analyzed SERS spectra of 60 acute promyelocytic leukemia cells (HL60 cell line), 60 chronic myelogenous leukemia cells (K562 cell line), and 60 normal human bone marrow mononuclear cells (Yu et al., 2017). The AgNPs were delivered into the cells by sonoporation. The results demonstrated that the intensity of the SERS band at 1005 cm^–1^ was highest in the case of the normal bone marrow mononuclear cells, followed by the chronic myelogenous leukemia cells, while the acute promyelocytic leukemia cells exhibited the least intense band at 1005 cm^–1^. We have recently seen a similar trend in the case of DNA extracted from the lymph nodes of patients with non-Hodgkin lymphoma and from control subjects: cancer DNA exhibited a decreased intensity in the SERS band at 1005 cm^–1^. Thus, there is a growing body of evidence suggesting a stepwise decrease in the intensity of SERS band at 1005 cm^–1^ of DNA that accompanies the onset and progression of cancer.

Compared to conventional techniques of analyzing DNA such as sequencing or ELISA, SERS has the potential to overcome many shortcomings, being a rapid and inexpensive alternative method that can be applied in the point-of-care setting. SERS can exploit the propensity of cancer DNA to adsorb onto metal nanoparticles, enabling the detection of cancer DNA even in the milieu of normal DNA, thus bypassing the need to isolate or purify cancer DNA.

An important limitation of this study is the relatively low number of samples, explained by the low incidence of DS-associated malignancies. Moreover, the study was performed retrospectively. Nonetheless, the results of this preliminary study suggest that SERS can detect the changes in the methylation landscape of DNA that accompanies the progression from a pre-leukemic to a leukemic state. It is expected that future improvements in the intrinsic prerogatives of the technique and the high degree of refinement in nano-manufacturing will translate into reliable and robust real-life applications in the near-future.

## Conclusion

The DNA SERS marker band at 1005 cm^–1^ points toward a stepwise decrease in DNA global methylation as cells progress from a pre-leukemia to a full-blown acute leukemia. In addition, this gradual decrease in DNA methylation occurs in both DS-associated malignancies and in patients without DS.

## Data Availability Statement

The original contributions presented in the study are included in the article/Supplementary Material, further inquiries can be directed to the corresponding authors.

## Ethics Statement

The studies involving human participants were reviewed and approved by Iuliu Hatieganu University, School of Doctoral Studies, as well as of the Emergency Clinical Children’s Hospital, both in Cluj-Napoca, Romania. Written informed consent to participate in this study was provided by the participants’ legal guardian/next of kin.

## Author Contributions

VM and VS: conceptualization, methodology, investigation, data analysis and interpretation, and writing – original draft. AS: conceptualization, methodology, investigation, data curation, and writing – review, and editing. SI: methodology, data curation, investigation, and writing – review and editing. AJ, SP, SI, A-AZ, AT, PT, CT, CB, DD, GP, SA, SM, and AC: methodology and validation. NL: writing – review and editing, project administration, and funding acquisition. CT: conceptualization, study approval, informed consent obtaining from all subjects, writing – review and editing, and funding acquisition. All authors contributed to the article and approved the submitted version.

## Conflict of Interest

The authors declare that the research was conducted in the absence of any commercial or financial relationships that could be construed as a potential conflict of interest.

## Publisher’s Note

All claims expressed in this article are solely those of the authors and do not necessarily represent those of their affiliated organizations, or those of the publisher, the editors and the reviewers. Any product that may be evaluated in this article, or claim that may be made by its manufacturer, is not guaranteed or endorsed by the publisher.

## Abbreviations

SERS: surface-enhanced Raman scattering
AL: acute leukemia
AL-DS: acute leukemia associated with down syndrome
TL-DS: transient leukemia associated with down syndrome
MPN: myeloproliferative neoplasm
Non-DS-MPN: non-down syndrome myeloproliferative neoplasm
Non-DS-AL: non-down syndrome acute leukemia.
