# Supplementary material for: SERS-Based Evaluation of the DNA Methylation Pattern Associated With Progression in Clonal Leukemogenesis of Down Syndrome
Source: Front Bioeng Biotechnol. 2021 Jul 23;9:703268. doi: 10.3389/fbioe.2021.703268 (PMC8343173; doi:10.3389/fbioe.2021.703268)
Supplement: Supplementary file 1 [file Data_Sheet_1.PDF]

Supplementary Information

SERS-based evaluation of the DNA methylation pattern associated with progression in clonal leukemogenesis of Down syndrome

Vlad Moisoiu[1,#], Valentina Sas[2,3,#], Andrei Stefancu[1,#], Stefania D. Iancu[1], Ancuta Jurj[4], Sergiu Pasca[2], Sabina Iluta[2], Alina-Andreea Zimta[5], Adrian B. Tigu[5], Patric Teodorescu[2], Cristina Turcas[2], Cristina Blag[2], Delia Dima[2], Gheorghe Popa[3], Smaranda Arghirescu[6,7], Sorin Man[3], Anca Colita[8,9], Nicolae Leopold[1,10*], Ciprian Tomuleasa[2,5,11*]

[1]Faculty of Physics, Babeș-Bolyai University, Cluj-Napoca, Romania

[2]Department of Hematology, Iuliu Hatieganu University of Medicine and Pharmacy Cluj-Napoca, Romania

[3]Department of Pediatrics, Iuliu Hatieganu University of Medicine and Pharmacy Cluj-Napoca, Romania

[4]Research Center for Functional Genomics and Translational Medicine, Iuliu Hatieganu University of Medicine and Pharmacy Cluj-Napoca, Romania

[5]Medfuture Research Center for Advanced Medicine, Iuliu Hatieganu University of Medicine and Pharmacy Cluj-Napoca, Romania

[6]Department of Pediatrics, Victor Babeș University of Medicine and Pharmacy, Timisoara, Romania

[7]Department of Pediatrics, Louis Turcanu Emergency Hospital for Children, Timisoara, Romania

[8]Department of Pediatrics, Carol Davila University of Medicine and Pharmacy, Bucharest, Romania

[9]Department of Pediatrics, Fundeni Clinical Institute, Bucharest, Romania

[10] Biomed Data Analytics SRL, Cluj-Napoca, Romania

[11]Department of Hematology, Ion Chiricuta Clinical Cancer Center, Cluj-Napoca, Romania

#these authors contributed equally should be considered first authors

Corresponding author: Nicolae Leopold, Ciprian Tomuleasa

Email: Nicolae.Leopold@ubbcluj.ro

Ciprian.Tomuleasa@umfcluj.ro

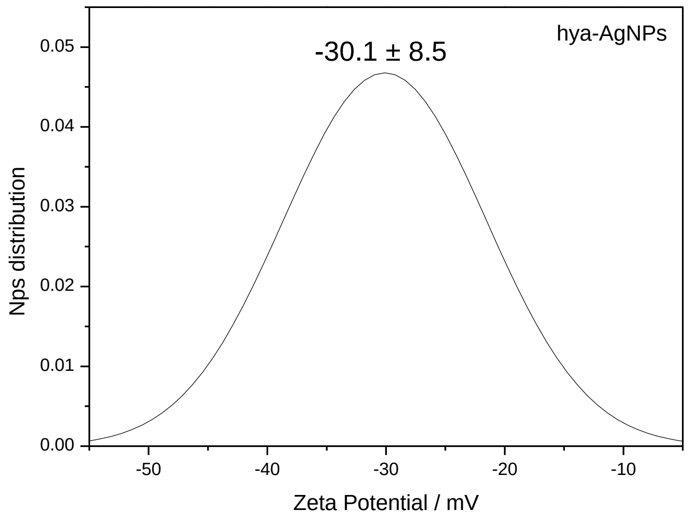

Figure S1. Zeta Potential of hya-AgNPs diluted ultrapure water, 1:1000 ratio.

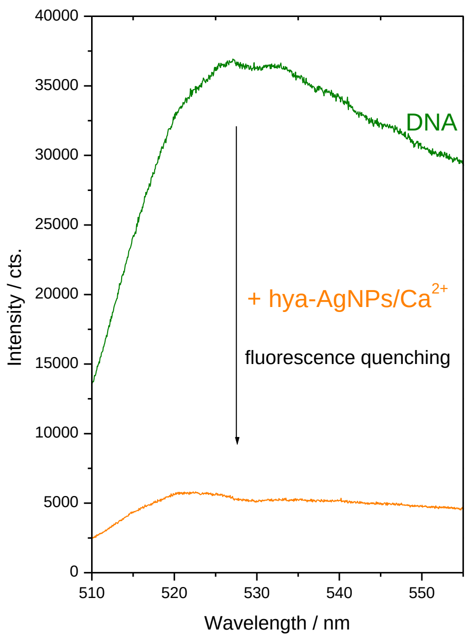

Figure S2. DNA adsorption on hya-AgNPs. The fluorescence emission spectra of Sybr green bound to DNA (green). The fluorescence is quenched when Sybr green-labeled DNA is adsorbed onto hya-AgNPs. A 442 nm laser line was used for sample excitation.

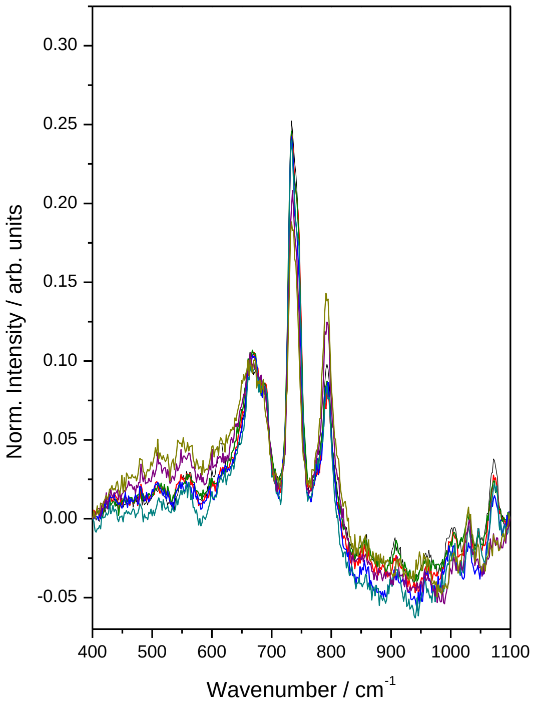

Figure S3. Reproducibility of the SERS spectra of DNA (150 ng/µl) extracted from a human cell line (HaCaT). The SERS spectra were acquired during an 8 hour period from 8 different mixtures of DNA and hya-AgNPs activated with Ca^{2+}.

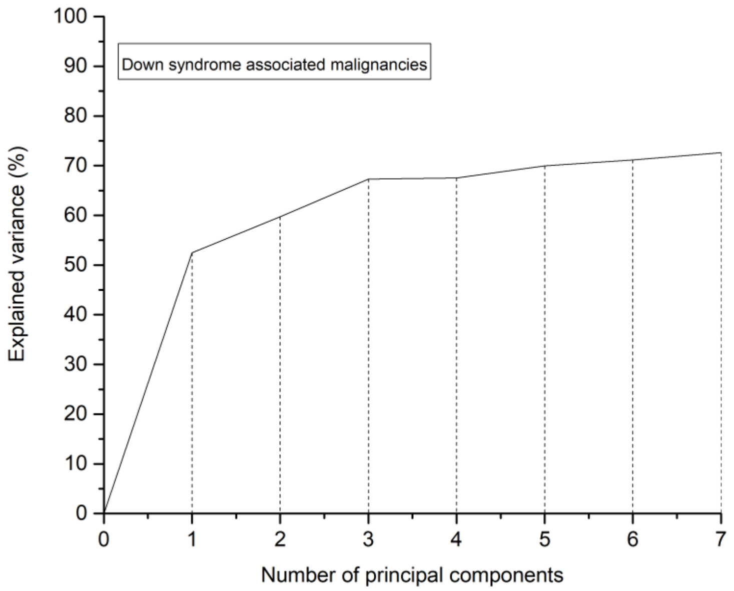

Figure S4. The explained variance of the principal components, obtained by principal component analysis (PCA) of the SERS spectra of DNA from patients with Down syndrome-associated malignancies.

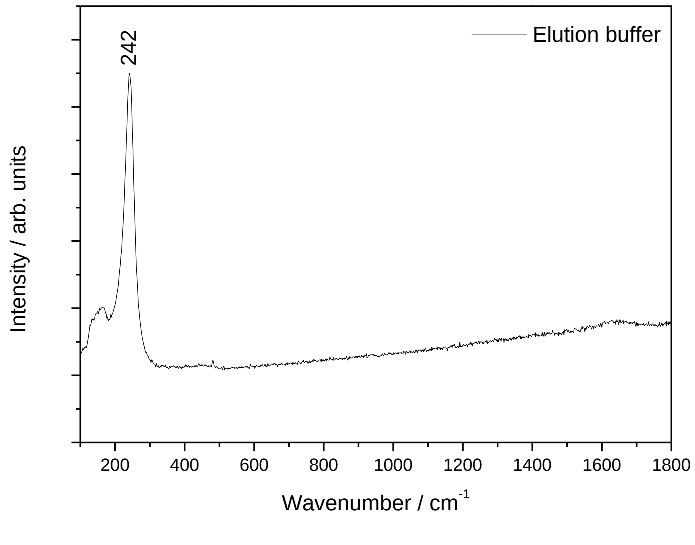

Figure S5. SERS spectrum of the Elution buffer used for DNA elution. The experimental conditions were the same as for the DNA SERS spectra acquisitions. The SERS band at 242 cm-1 is attributed to Ag-Cl vibration.

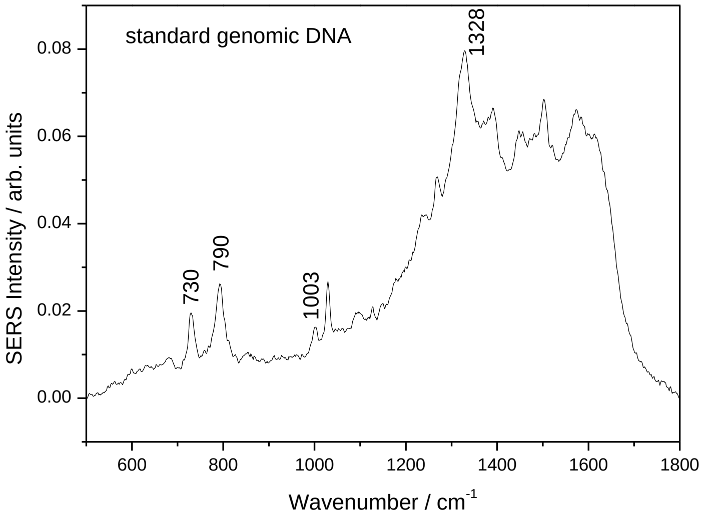

Figure S6. SERS spectrum of 100 ng/µl standard genomic DNA. The experimental conditions were the same as for the DNA SERS spectra acquisitions.

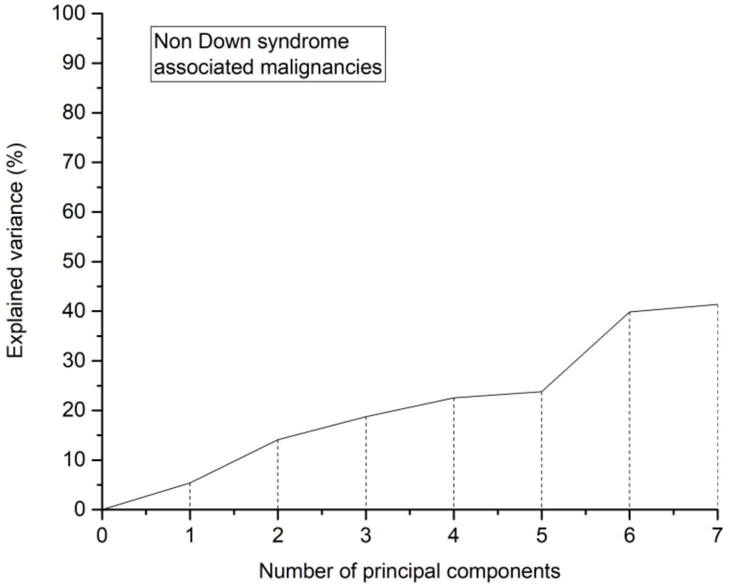

Figure S7. The explained variance of the principal components, obtained by principal component analysis (PCA) of the SERS spectra of DNA from patients with non-Down syndrome-associated malignancies.
